# Healthcare Utilization in a Large Cohort of Asylum Seekers Entering Western Europe in 2015

**DOI:** 10.3390/ijerph15102163

**Published:** 2018-10-01

**Authors:** Martin Wetzke, Christine Happle, Annabelle Vakilzadeh, Diana Ernst, Georgios Sogkas, Reinhold E. Schmidt, Georg M. N. Behrens, Christian Dopfer, Alexandra Jablonka

**Affiliations:** 1 Department of Pediatric Pneumology, Allergology, and Neonatology, Hannover Medical School, 30625 Hannover, Germany; wetzke.martin@mh-hannover.de (M.W.); happle.christine@mh-hannover.de (C.H.); dopfer.christian@mh-hannover.de (C.D.); 2 German Center for Infection Research (DZIF), Partner Site Hannover-Braunschweig, 38124 Braunschweig, Germany; Schmidt.Reinhold.Ernst@mh-hannover.de (R.E.S.); behrens.georg@mh-hannover.de (G.M.N.B.); 3 German Center for Lung Research, Biomedical Research in End Stage and Obstructive Lung Disease/BREATH Hannover, 30625 Hannover, Germany; 4 Hannover Medical School, 30625 Hannover, Germany; Annabelle.Schaell@stud.mh-hannover.de; 5 Department of Clinical Immunology and Rheumatology, Hannover Medical School, 30625 Hannover, Germany; ernst.diana@mh-hannover.de (D.E.); sogkas.georgios@mh-hannover.de (G.S.)

**Keywords:** healthcare, migration, refugee, asylum seeker, medical service, migrant, medical care, doctor, Europe, Germany

## Abstract

During the current period of immigration to Western Europe, national healthcare systems are confronted with high numbers of asylum seekers with largely unknown health status. To improve care taking strategies, we assessed healthcare utilization in a large, representative cohort of newly arriving migrants consisting of *n* = 1533 residents of a reception center in Northern Germany in 2015. Most asylum seekers were young, male adults, and the majority came from the Eastern Mediterranean region. Overall, we observed a frequency of 0.03 visits to the onsite primary healthcare ward per asylum seeker and day of camp residence (IQR 0.0–0.07, median duration of residence 38.0 days, IQR 30.0–54.25). Female asylum seekers showed higher healthcare utilization rates than their male counterparts, and healthcare utilization was particularly low in asylum seekers in their second decade of life. Furthermore, a significant correlation between time after camp entrance and healthcare utilization behavior occurred: During the first week of camp residence, 37.1 visits/100 asylum seekers were observed, opposed to only 9.5 visits/100 asylum seekers during the sixth week of camp residence. This first data on healthcare utilization in a large, representative asylum seeker cohort entering Western Europe during the current crisis shows that primary care is most needed in the first period directly after arrival. Our dataset may help to raise awareness for refugee and migrant healthcare needs and to adapt care taking strategies accordingly.

## 1. Introduction

At the height of the current refugee and migrant crisis, in 2015, more than 24 million migrants were on the move worldwide. Many of them entered Western Europe, with Germany being the leading destination for asylum seekers from countries such as Syria, Afghanistan, Somalia and others [1]. Appropriate medical care for this growing population of refugees and migrants represents an enormous challenge to the receiving population. During their migration, the vast majority of refugees and migrants had only irregular access to medical services and good sanitation, leaving them at high risk of communicable and non-communicable diseases [2,3,4,5,6]. Many refugees have experienced political and personal trauma such as war, detainment, torture, forced migration, and separation or death from family members and friends [7,8,9]. Depending on gender, age, or nationality, refugees may have different health status and health-seeking behaviors [10]. Although the current extent of immigration to Western Europe is considered to be the largest of our generation thus far [11], national healthcare systems across Europe are still struggling to provide appropriate care and meet the needs of immigrating refugees and migrants. Especially in the summer of 2015, when Western European countries were first confronted with a massive influx of persons with unclear health status and healthcare needs [12], national caregivers were comparably ill-prepared. It took a long time to harmonize efforts for the set-up of primary care structures that were effective, cost-sensitive and, most importantly, met the refugees’ and migrants’ needs [13,14]. However, there is still little knowledge on general healthcare utilization among refugees and migrants during the current crisis, and updated information on the specific demand for emergency onsite healthcare in refugee camps is scarce. To treat and prevent further disease burden in this vulnerable population, and to improve primary care in extreme humanitarian situations similar to the current situation, we aimed to analyze primary healthcare utilization behavior in a well characterized, representative cohort of asylum seekers in a Northern German refugee camp during the current exodus.

## 2. Methods

### 2.1. Study Population

For the description of healthcare utilization in asylum seekers, data on all residents (*n* = 1533) of a reception center in Celle, Northern Germany in September–December 2015 were analyzed. All subjects had been allocated to Lower Saxony based on a federal state specific German allocation key (Königssteiner Schlüssel) and were sent to a reception center in Celle based on space availability. They were not preselected in any fashion. All residents were asylum seekers and registered upon arrival and their departure date was documented. For asylum seekers leaving the center without notice to camp authorities, last contact documentation of the camp staff (for example, medical service, food service, transportation) was used as date of departure. Parts of this cohort have been described previously [15,16,17].

### 2.2. Medical Service and Analysis of Healthcare Utilization

A 24 h-onsite medical ward was available to all residents of the center. This ward offered medical services at primary medical care level with constant paramedical emergency service and daily consultation hours by medical doctors for general medical treatment. For medical problems exceeding general medical care, further referral to specialists was arranged whenever needed. All visits to the onsite medical center were documented in electronic form. Mandatory primary check-ups of asylum seekers upon arrival, as required by asylum law, were not included in the data set for this analysis. Health care utilization was determined by analyzing individual presentations to the onsite medical ward in relation to personal days of residence at the reception center.

### 2.3. Data Analysis

All information was collected in routine clinical care. Proband specific information on age, gender, country of origin, attendance dates, and visits to the medical center was extracted from an electronic database. All data had been fully anonymized by the Order of Malta (Malteser Hilfsdienst) before scientific analysis.

### 2.4. Statistics

Analyses were conducted employing SPSS version 24.0 (IBM, Armonk, NY, USA) and Graphpad Prism version 5.02 (GraphPad Software, La Jolla, CA, USA). Descriptive statistics were assessed using median and range or interquartile range (IQR) for non-normally distributed variables and using mean ± SD for normally distributed variables or a combination of both. Group differences with categorical items were evaluated by Mann-Whitney-U or one-way ANOVA/Kruskal-Wallis testing and *p* values below 0.05 were considered significant.

### 2.5. Ethics Compliance

All analyses were approved by local authorities (Institutional Review Board of Hannover Medical School approval #2972-2015). All patient information was anonymized prior to analysis.

## 3. Results

**Figure 1:**
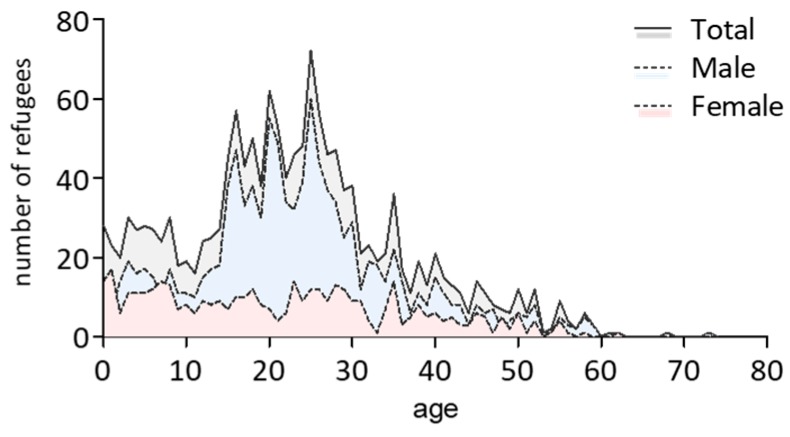
Age and gender distribution within the analyzed cohort.

Data of healthcare utilization of *n* = 1533 asylum seekers was included in the analysis. For *n* = 45 asylum seekers, information on nationality and for *n* = 36, information on age was missing. Overall, 71.8% of asylum seekers were male. The median age of all probands was 22 years (male asylum seekers 23 years, range 0–73 years; female asylum seekers 21 years, range 0–62 years, Figure 1). The vast majority of probands (80.5%) came from the Eastern Mediterranean region, followed by two smaller proportions from Europe (7.7%) and Africa (7.7%) and only few persons from Southeast Asia (1.2%) or of unknown origin (2.9%).

**Figure 2:**
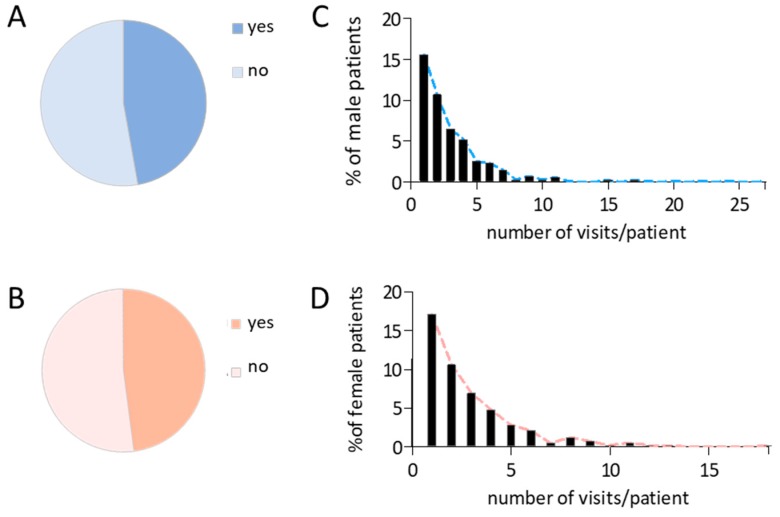
Healthcare utilization within the analyzed refugee cohort. (**A**,**B**) Proportion of male and female probands seeking help (yes) or not (no) in the medical ward. (**C**,**D**) visit number distribution in male and female patients [HCU: healthcare utilization].

During their stay at the reception center, overall 47.4% of the asylum seekers utilized medical care at the ward. 47.2% of male refugees and 48% of female refugees presented at the primary healthcare unit of the shelter (Figure 2A,B). Out of all patients that visited the primary healthcare unit, male patients sought medical help up to 27 times (median 2 (QR 1–4), mean 3.2 ± SD 3.1), and female patients paid up to 18 visits to the onsite clinical team (median 2 (IQR 1–4), mean 2.9 ±SD 2.5), Figure 2C,D). Irrespective of gender, the vast majority of patients paid between 1 and 5 visits to the medical ward, and the median visit number was 2 (IQR 1–4, mean 3.1 ± SD 3.0) visits per patient.

**Figure 3:**
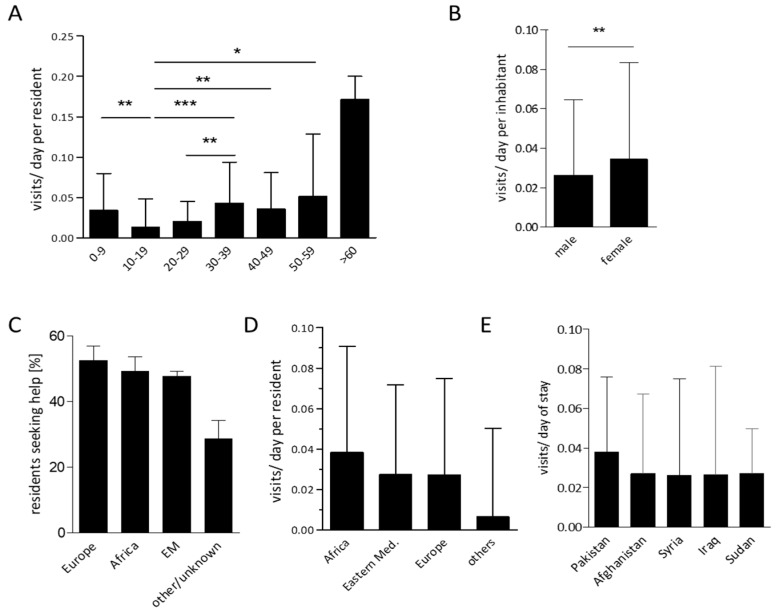
Factors influencing healthcare utilization in the analyzed cohort. Visits to the onsite medical ward per day of camp residence in age specific subgroups (**A**) and males vs. females (**B**). Percentage of asylum seekers utilizing medical help amongst all camp residents from this WHO region of origin (**C**). Medical consultations per day of camp residence in asylum seekers from different regions of origin (**D**) or from the top five most prevalent nations within the cohort (**E**) (bars display mean + IQR (A,B,D,E) and mean ± 95%CI (C), * *p* ≤ 0.05, ** *p* ≤ 0.01, *** *p* ≤ 0.005).

Next, we analyzed individual factors possibly influencing healthcare utilization in asylum seekers. For *n* = 1094 asylum seekers, exact entrance and exit dates of camp residence were available. They had a median duration of camp inhabitation of 38.0 (IQR 30–54.25, mean 41.3 ± 0.7) days. We observed a median visit frequency of 0.03 per asylum seeker and day of camp residence (IQR 0.0–0.07). The highest rate of healthcare utilization occurred in asylum seekers above the age of 60 years, and a particularly low rate of medical visits per day in the age group of 10–19 years in our cohort was found. Children below the age of ten years and adults in their fourth, fifth and sixth decade of life showed significantly higher healthcare utilization rates. In addition, young adults aged 20–29 years spent significantly less visits to the onsite medical ward than those aged 30–39 years (Figure 3A). When we compared male and female asylum seekers in our cohort, we found a significantly higher rate of healthcare utilization in females (Figure 3B). With regard to the asylum seekers’ origins, no significant influence on health care utilization behavior was observed: WHO regions of origin had no significant effect on the percentage of refugees seeking help at the onsite ward, and were also not associated with significantly different frequencies of healthcare utilization per day of camp residence (Figure 3C,D, Table S1). When focusing on the top five most prevalent nationalities within the cohort, the frequency of visits per day were also not significantly different between asylum seekers from Afghanistan, Syria, Iraq, Pakistan, or Sudan (Figure 3E). For relative visit numbers from other nations of this cohort, please refer to Supplementary Figure S1.

**Figure 4:**
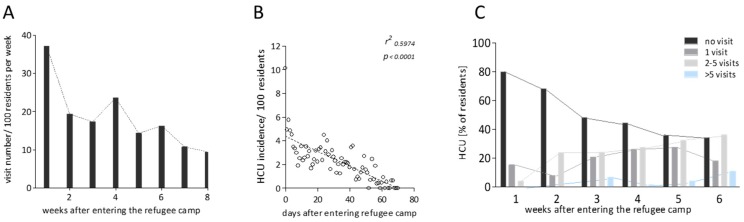
Time of refugee center inhabitance influences health care utilization. (**A**) Healthcare utilization rates per 100 camp residents per week after entering the shelter. (**B**) Correlation of healthcare utilization rates per 100 residents with residence duration in days. (**C**) Overall healthcare utilization depending on personal week of stay at the center (HCU: healthcare utilization).

The timepoint of stay at the camp had clear influence on the probability of overall healthcare utilization within the cohort. Most patients visited the medical ward during their first week of stay, and fewest during their last week of camp inhabitation (Figure 4A). During the first week of personal camp residence, 37.1 visits per 100 asylum seekers occurred, whereas only 9.5 visits per 100 asylum seekers were noted in week six of inhabitance at the reception center. A significant correlation between duration of camp residence in days and relative number of visits occurred, with clearly higher rates of healthcare utilization on the day of arrival at the camp (10.1 visits per 100 residents) compared to only 0.1 visits per 100 residents on day 67 and no visits on day 70 of personal camp inhabitance (Figure 4B). Overall, the proportion of asylum seekers that had not sought medical help at the reception center at all declined over time, whilst the percentage of probands with one, two to five or more than five visits gradually increased (Figure 4C).

## 4. Discussion

To the best of our knowledge, we present here the first comprehensive data on age, gender and origin dependent healthcare utilization behavior in a large cohort of newly arriving asylum seekers in Western Europe during the current refugee and migrant crisis. The fact that the largest proportion of asylum seekers in our cohort were young, male adults and many of them came from the Eastern Mediterranean region is in accordance with current pan-European immigration statistics [18,19,20].

Primary medical service for refugees and migrants is a corner stone of humanitarian care during the current crisis [11]. Political conflicts and wars in the Middle East and Africa as well as economic imbalances in Eastern Europe and many other parts of the world have led to extensive migration towards Western Europe. In 2015, more than 24 million people worldwide were fleeing, and Germany represented a top destination for many asylum seekers [1]. As such, the German healthcare system has been particularly challenged in taking primary care of newly arriving asylum seekers of unknown health status [1,12,21]. Despite this enormous challenge—which is experienced by most healthcare systems in Europe—primary healthcare needs of arriving immigrants has thus far received little attention. This is a dilemma, as provision of easily accessible healthcare to asylum seekers upon arrival, on transit or even for longer periods at onsite medical wards is essential to appropriately manage this humanitarian situation [11].

In this context, we aimed to compile an initial data set describing the health care utilization experience after setting up onsite primary medical care at an asylum seeker reception center in Northern Germany in 2015. In our cohort, a high percentage of asylum seekers sought onsite medical help, with almost half of all migrants seeking medical exceeding the initial mandatory checkup visit. The time after entering the refugee camp appeared to have a strong effect on personal healthcare utilization. An inverse correlation of visit frequency with days of camp inhabitance occurred, and the probability of presentation at the medical ward was significantly higher in the first compared to the last day of camp residence. In the first week after entering the refugee camp, more than 37 visits per 100 camp residents were noted, whereas during week five and six of camp inhabitation, only 10.8 and 9.45 visits per 100 asylum seekers occurred. This observation may have significant impact on the planning of primary healthcare setup in future situations of similar nature.

Our data clearly shows that primary medical care is most needed when a group of asylum seekers arrive, and it could make sense to intensify personnel and medical supplies for an onsite medical ward in preparation for newly arriving cohorts. Our data supports the notion that, after an initial rush of patients (in our cohort around 10 visits per day and 100 asylum seekers on the day of camp entry), the need for primary care gradually declines (in our cohort 75% reduction with 2.25 visits per 100 migrants after around one month).

Of note, the visit frequency described in our cohort was much higher than that described by Hermans et al. in their brief report on healthcare utilization at two Greek refugee sites: here the authors observed only 3.6 patient visits per person years in *n* = 298 newly arriving refugees in Lesbos [22]. We can only speculate on why rates were much lower in this Greek cohort and assume that different study design, primary care accessibility, overall organization in the reception centers, and possibly other factors such as nationality were considerably different from our setting.

However, certain limitations need to be taken into account when interpreting our dataset. Our results cannot be extrapolated too far, as the current refugee situation is complex and our cohort, albeit representative according to overall demographic data, is just a small specimen of the large extent of migrants currently entering Western Europe. Furthermore, this cohort is unique with regard to the fact that onsite general practitioner care was offered on a daily basis and that specialists (e.g., pediatricians, surgeons, psychiatrists) were available for referral. An interpreter service was available whenever needed and present for most consultations. All medical appointments as well as medication were free of charge. These structural circumstances were designed to facilitate easy access to care and may therefore have resulted in rather high utilization. We used this dataset to describe refugee healthcare utilization, because the underlying cohort constituted of a large and unselected specimen of newly arriving asylum seekers with well-documented inhabitance duration at the initial migrant residence in Germany. The provision of full time primary medical care at an onsite ward of the asylum seeker residence—with patient specific documentation of visit time and frequency—was unique to the here presented cohort and ideal for the conducted analysis. In other cohorts thus far analyzed by our team, similar analysis is hampered by limited data extraction of residence time and medical records that could only be obtained by a proportion of asylum seekers or were collected in a different fashion than in the here described cohort [15,16,17,19,20,23,24]. Currently, however, we aim at harmonizing and analyzing further large asylum seeker cohorts of a similar kind to see whether our results can be confirmed in a greater specimen of migrants entering Western Europe during the current crisis. For example, our observed statistical independence of country of origin from healthcare seeking behavior may very well be based on the low number of probands from single countries. This prohibits comprehensive statistical analyses and should be further analyzed in larger cohorts or meta-analyses of probands with several thousand migrants thus far not available to us. 

Refugees and migrants are not a homogenous population, and depending on home countries, personal history, motivation to emigrate, and experiences of violence and neglect, the medical needs of persons on the run may vary considerably [22,23,25]. In addition, age is a clear factor influencing an asylum seeker’s medical needs, with children being particularly vulnerable to neglect and insufficient healthcare [19,26,27]. Indeed, we found higher healthcare utilization rates in young children below the age of ten years compared to older children, adolescents and young adults and observed the highest rate of medical demand in the oldest refugee group above the age of 60 years. Furthermore, women in our cohort displayed higher healthcare utilization rates than males. These findings are in line with previous reports on the high risk of elder refugees and females for increased migration associated morbidity [1,5]. Our future analyses will focus on pediatric primary healthcare utilization and age-dependent complaints in different refugee and migrant cohorts.

Taken together, this data emphasize the high demand of migrants for primary care when first arriving to a destination country with high socioeconomic standard and stress their strong need for high accessibility and affordability of health services during the first period after arrival. As such, our data may help to plan and organize primary healthcare facilities for arriving migrants in countries with high economic resources according to the patient needs.

The UNHCR has recently stated that optimal refugee care during the current humanitarian crisis demands a “multidimensional and comprehensive approach in public health and nutrition, and will require funding and donations of both technical support and commodities/funds beyond the normal programming needs” [28]. The European Commission has recognized the demand for structured analysis of refugee medical needs and setup the “3rd Health Programme by the Consumers, Health, Agriculture and Food Executive Agency (CHAFEA)” to fund projects aiming at assessing healthcare needs of refugees reaching Europe, and development, improvement, and testing of health educational tools for this population [11].

The growing population of refugees and migrants currently entering Europe poses enormous challenges to the receiving communities and their healthcare systems and, in accordance with the UNHCR vision and the World Health Organization, requires effective onsite healthcare programs based on reliable epidemiological data [29]. In this context, we would like to emphasize the particular need for primary care demand in newly arriving refugees and migrants and hope that our initial analysis helps to better estimate needs in the vulnerable migrating population, and to adapt care taking strategies accordingly.

## Supplementary Materials

The following are available online at http://www.mdpi.com/1660-4601/15/10/2163/s1. Figure S1: Visits per day of refugee center inhabitance depending on country of origin [median ± IQR], Table S1: WHO regions of origin.

## Abbreviations

CI	confidence interval
HCU	healthcare utilization
IQR	interquartile range
SEM	standard error mean
WHO	world health organization
