# Supplementary material for: Healthcare Utilization in a Large Cohort of Asylum Seekers Entering Western Europe in 2015
Source: Int J Environ Res Public Health. 2018 Oct 1;15(10):2163. doi: 10.3390/ijerph15102163 (PMC6210699; doi:10.3390/ijerph15102163)
Supplement: Supplementary file 1 [file ijerph-15-02163-s001.pdf]

International Journal of
Environmental Research and Public Health

MDPI

Supplementary Materials

Healthcare utilization in a large cohort of refugees entering Western Europe in 2015

Martin Wetzke, Christine Happle, Annabelle Vakilzadeh, Diana Ernst, Georgios Sogkas, Reinhold E. Schmidt, Georg M. N. Behrens, Christian Dopfer and Alexandra Jablonka

Table S1. WHO regions of origin.

European Region	Eastern Mediterranen Region	African Region	South East Asia
Albania	Afghanistan	Algeria	Bangladesh
Armenia	Egypt	Ivory Coast	
Azerbaijan	Iraq	Eritrea	
Bosnia	Iran	Ghana	
Georgia	Libya	Mali	
Kosovo	Morocco	Nigeria	
Montenegro	Pakistan	Somalia	
Serbia	Palestine	Sudan	
Ukraine	Syria		
	Tunesia		

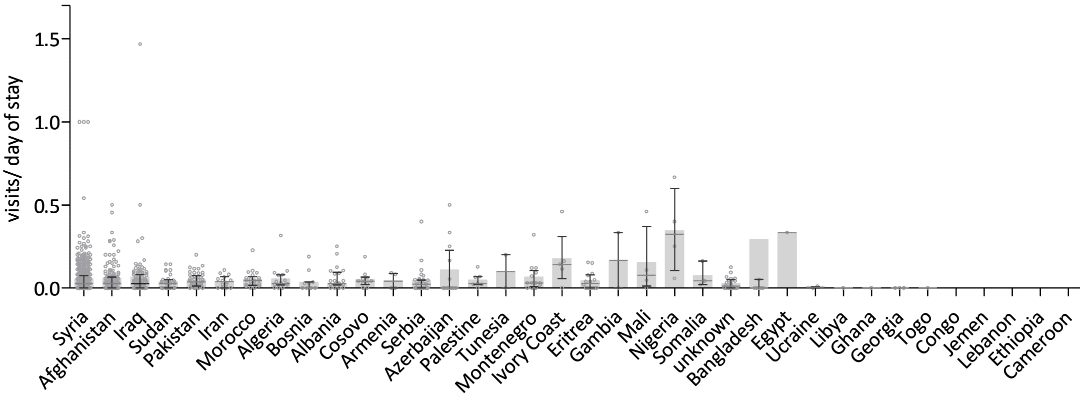

Figure S1. Visits per day of refugee center inhabitance depending on country of origin [median ± IQR].
